# Establishment of a CRISPR/Cas9-based strategy for inducible protein dimerization

**DOI:** 10.17912/W2208R

**Published:** 2018-04-17

**Authors:** Jeffrey Zielich, Sriyash Mangal, Esther Zanin, Eric J. Lambie

**Affiliations:** 1 Department of Cell and Developmental Biology, Ludwig-Maximillians-University, Munich, Planegg-Martinsried, Germany.

**Figure 1: CRISPR-based dual-marker selection cassette for rapamycin-induced protein dimerization in C. elegans.**
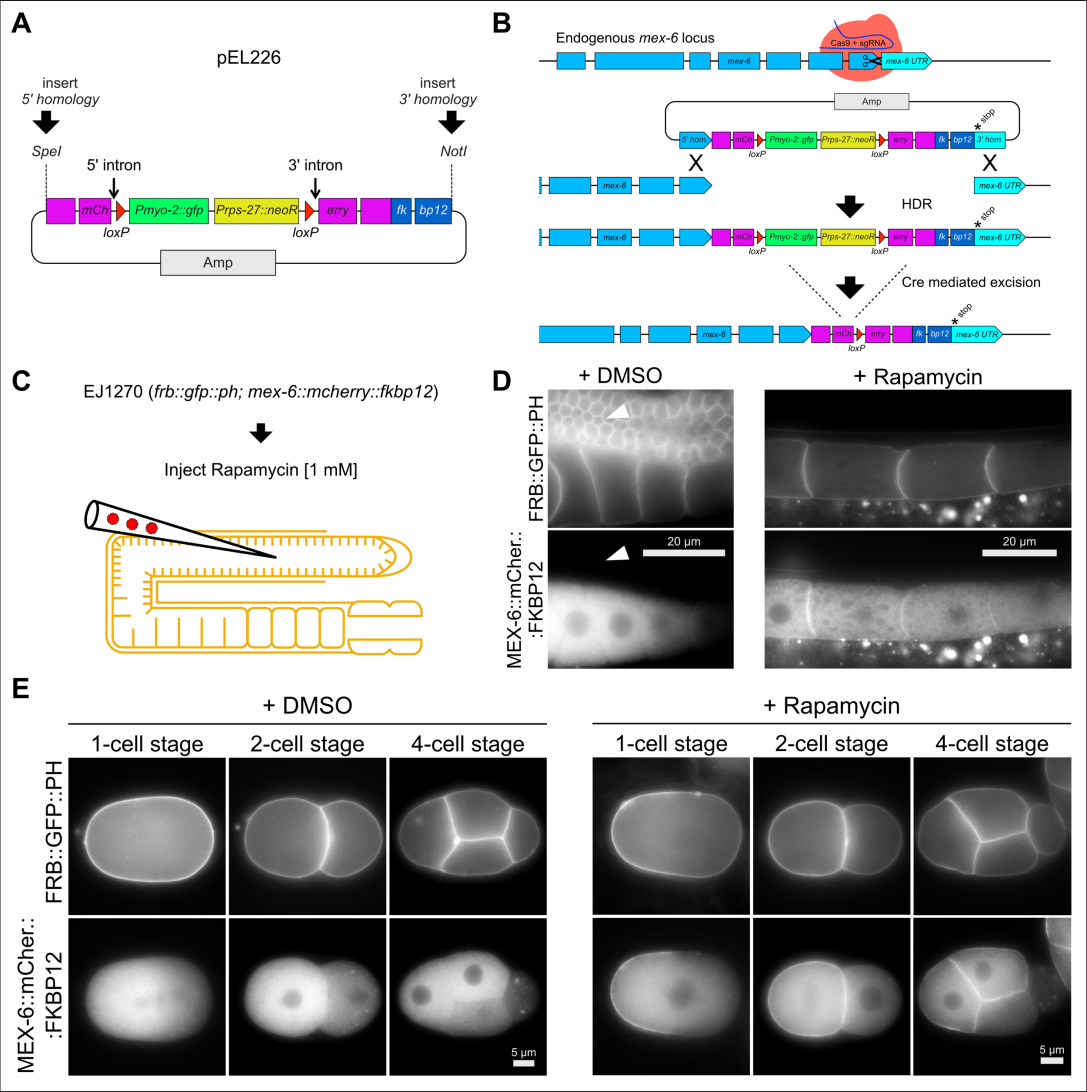
(A) The codon-optimized *fkbp12* sequence together with a *NotI* site for 3’ homology region insertion was introduced in the *mCherry*-tag repair donor vector (Norris *et al.* 2015) to generate vector pEL226. (B) Schematic overview of of CRISPR/Cas9 mediated *mcherry::fkbp12* tag of endogenous *mex-6*, including Cre-mediated excision of the dual marker selection cassette, resulting in strain EJ1269. (C) Injection of 1 mM rapamycin into adult stage gonads (Mangal *et al.* 2018) of strain EJ1270 expressing FRB::GFP::PH and MEX-6::mCherry::FKBP12 (obtained by crossing EJ1269 into ZAN87, Table **2**). (D) Epifluorescence microscopy images of adult stage proximal gonads imaged ~ 6 hours after the gonads were injected with DMSO (*n* = 4) or 1 mM rapamycin (*n* = 5). MEX-6::mCherry::FKBP12 signal increases in later stage oocytes and remains cytosolic if 10 % DMSO is injected into the gonad. Arrowheads point to the pachytene region of the gonad where MEX-6::mCherry::FKBP12 is not detectable. Injection of rapamycin induces the binding of the FRB and FKBP12 domains and thereby translocates MEX-6::mCherry::FKBP12 to the plasma membrane of diakinesis-stage oocytes. (E) Epifluorescence microscopy images of 1-cell, 2-cell and 4-cell stage embryos imaged ~ 6 hours after the gonads were injected with DMSO (1-cell stage *n* = 5; 2-cell stage *n* = 4; 4-cell stage *n =* 7) or 1 mM rapamycin (1-cell stage *n* = 10; 2-cell stage *n* = 16; 4 cell stage *n =* 10). 1-cell stage embryos showing a cytosolic anterior gradient and 2-cell / 4-cell stage embryos showing strong expression in the AB lineage (DMSO). Upon rapamycin injection, MEX-6::mCherry::FKBP12 translocates to the plasma membrane in the anterior region (1-cell stage) and the anterior blastomeres (2-cell and 4-cell stage).

## Description

Induced protein dimerization is a useful tool to study protein function. A well-established method takes advantage of the binding between the FKBP12 protein (FK506 binding protein 12 kDa) and the FRB domain of the mTOR kinase upon interaction with rapamycin (Putyrski and Schultz 2012). Recently we established a rapamycin-inducible dimerization system for the germ line and early embryos of *C. elegans* (Mangal *et al.* 2018). We demonstrated the translocation of mCherry::FKBP12 to the plasma membrane via rapamycin induced binding to FRB::GFP::PH (anchored to the plasma membrane). In order to study the function of a native protein upon rapamycin induced translocation it would be advantageous to tag the genomic region of the gene of interest (GOI) with *mCherry::fkbp2* to ensure that the fusion protein is controlled by its native environment.

The CRISPR/Cas9 system has revolutionized genome engineering in *C. elegans* (Chen *et al.* 2013; Arribere *et al.*2014; Paix *et al.* 2015; Dickinson *et al.* 2015; Norris *et al.* 2015; Schwartz and Jorgensen 2016). DNA double-stranded breaks are generated by the endonuclease Cas9, which is guided to its target by a single guide RNA (sgRNA) (Jinek *et al.* 2012). If a repair donor vector is provided carrying a transgenic sequence flanked by 5’ and 3’ homology regions, the cell can repair these double-stranded breaks via homology directed repair (HDR) by incorporating the transgenic sequence into the cleaved locus. This enables *e*.*g*. N- or C-terminal fluorescent protein fusions of the GOI. Drug-selection based screening methods to tag native proteins have been developed (Dickinson *et al.* 2015; Norris *et al.* 2015; Schwartz and Jorgensen 2016). One of these streamlined methods uses a dual marker selection cassette (Norris *et al.* 2015).

Herein, we describe a modification of the dual-marker selection cassette plasmid of Norris *et al*. (2015) that can be used in conjunction with CRISPR/Cas9, TALEN or Zinc Finger Nucleases to tag endogenous proteins for inducible translocation to the plasma membrane during early embryogenesis or in the germ line.

We modified Norris’s *mCherry*-tag repair donor vector by fusing a *C. elegans* codon-optimized *fkbp12* sequence 3’ to *mCherry* (Mangal *et al.* 2018) and re-establishing the critical *NotI* site for 3’ homology region insertion (Figure 1 A; pEL226). This vector can be used to C-terminally tag any GOI by following the protocol of Norris *et al*. (2015), including subsequent Cre-mediated excision of the dual marker selection cassette (Figure 1 B). As proof of principle we C-terminally tagged the locus of *mex-6* on chromosome II with *mCherry::fkbp12* (Figure 1 B). We observed increasing cytoplasmic mCherry signal in late-stage oocytes, and anterior enrichment in early-stage embryos (Figure 1 D and E; DMSO control). We did not detect any mCherry signal in the pachytene region of adult stage gonads (Figure 1 D arrowheads; DMSO control). These observations confirm previous localization studies for *mex-6(ax2065*[*mex-6::gfp*]*)* II transgenic animals (Paix *et al.* 2014), and mirror those for observed for the paralogous protein, MEX-5 (Schubert *et al.* 2000; Griffin *et al.* 2011). We crossed the MEX-6::mCherry::FKBP12 expressing strain (EJ1269, Table 2) with a strain that expresses FRB::GFP::PH (ZAN87; Mangal *et al.,* 2018), which localizes to the plasma membrane in the germ line and early embryos (Figure 1 D and E), and then singled F2 hermaphrodites to obtain a strain that is homozygous for both insertions (EJ1270, Table 2). Animals from this strain were injected with 1 mM rapamycin into the pachytene region of the germ line to induce binding between the FRB and FKBP12 domains. As expected, we observed strong accumulation of MEX-6::mCherry::FKBP12 signal at the plasma membrane of late-stage oocytes and anterior blastomeres of early embryos, 6 h after injection (Figure 1 D and E). Importantly, the MEX-6::mCherry::FKBP12 remains cytoplasmic if DMSO is injected into the germ line as a control (Figure 1 D and E).

Our repair donor vector can be easily modified to tag any GOI with *mCherry::fkbp12* in a well-established and streamlined manner (Norris *et al.* 2015) and it expands the *C. elegans* CRISPR/Cas9 toolbox for the rapamycin-inducible dimerization system. By crossing into the strain that expresses FRB::GFP::PH (ZAN87) (Mangal *et al.* 2018) it becomes possible to translocate an endogenously tagged mCherry::FKBP12 protein to the plasma membrane of early embryos or the germ line. Additionally, MEX-6::mCherry::FKBP12 could be used to enrich a protein-of-interest that is tagged with FRB::GFP within the anterior region of early embryos. 2018) it becomes possible to translocate an endogenously tagged mCherry::FKBP12 protein to the plasma membrane of early embryos or the germ line. Additionally, MEX-6::mCherry::FKBP12 could be used to enrich a protein-of-interest that is tagged with FRB::GFP within the anterior region of early embryos.

## Reagents

Standard methods for DNA amplification, analysis and manipulation were used. PCR products were amplified by using Phusion® High-Fidelity DNA Polymerase (New England Biolabs), according to the manufacturer’s protocol. DNA sequences were obtained by Sanger sequencing.

We inserted a *C. elegans* codon optimized sequence of *fkbp12* (plus a flexible linker 5’ of *fkbp12*, gcaggtggaggtact) into the unique *NotI* site of loxP_myo2_neoR_mCherry_intron (Norris *et al.* 2015) via Gibson assembly (Gibson *et al.* 2009) by creating a new unique *NotI* site 3’ of *fkbp12*. The resulting vector pEL226 is a universal repair donor vector to tag any locus with *mCherry::fkbp12*. We digested pEL226 with *SpeI* and *NotI* and subsequently column purified the DNA. We amplified ~575 bp 5’ and 3’ homology region of the C-terminus of *mex-6* from genomic DNA (3’ homology region includes the stop codon of *mex-6* exon7, PAM(mex-6.1) was mutated to NCA) with primers carrying homology tails (Table 1) to the unique *SpeI* and *NotI* sites of pEL226. PCR products were subsequently column purified. All four fragments (PCR products plus pEL226 fragments) were fused together via Gibson assembly (pEL228).

We designed sgRNA(mex-6.1) via the online sgRNA design tool http://crispr.cos.uni-heidelberg.de/index.html(Stemmer *et al.* 2015). sgRNA(mex-6.1) is almost identical to sgRNA18 (Paix *et al.* 2014) but shifted by 1 nt 3’ and therefore using another PAM. sgRNA(mex-6.1) expression plasmid (pEL227) was cloned by using pRB1017 following the protocol of (Arribere *et al.* 2014). All plasmids were purified by using PureLink™ HQ Mini Plasmid DNA Purification Kit (Invitrogen) and eluted with Ultrapure Water for Molecular Biology (Merck / EMD MilliporeSigma). We used the following injection-mix concentrations (Norris *et al.* 2015): Peft-3::Cas9_SV40_NLS::tbb-2_UTR (*Peft-3Cas9* expression plasmid) at 50 ng/µl, sgRNA(mex-6.1) plasmid (pEL227, based on pRB1017) at 100 ng/µl, repair donor vector (pEL228) at 50 ng/µl (based on pEL226), pCFJ90 (Frøkjaer-Jensen *et al.* 2014) at 2.5 ng/µl (*Pmyo-2mcherry*), pCFJ104 (Frøkjaer-Jensen *et al.* 2014) at 5ng/µl (*Pmyo-3mcherry*). Ultrapure Water for Molecular Biology (Merck / EMD MilliporeSigma) was added to a total volume of 10 µl.

We injected 62 young adults (N2) and obtained 7 independent lines. We proceeded with one line and injected a Cre recombinase expression vector (pDD104). We used the following injection-mix concentrations: pDD104 (Dickinson *et al.* 2013) at 50 ng/µl and pCFJ90 at 2.5 ng/µl. After successful Cre-mediated excision of the dual marker selection cassette (Norris *et al.* 2015), we obtained the strain EJ1269 (Table 2) showing mCherry signal consistent with GFP signal observed in *mex-6(ax2065*[*mex-6::gfp*]*)* II (Paix *et al.* 2014). Finally we crossed EJ1269 into ZAN87 (Mangal *et al.* 2018) resulting in EJ1270 (Table 2).

The rapamycin-induced dimerization was performed according to (Mangal *et al.* 2018). Microscopy was performed 6 h after injection. Worms were dissected with 20 gauge hypodermic needles in M9 to release embryos. Whole worms were immobilized with 10 mM levamisole. Whole worms or embryos and were mounted on 4% agarose pads. Animals were imaged by using Zeiss Axioskop 2 and MetaMorph software (Molecular Devices). Image processing was performed in Fiji/imageJ 2.0.0 (Schindelin *et al.* 2012), brightness and contrast were adjusted either in Fiji/imageJ 2.0.0 or Affinity Designer 1.6.1 (Serif).

​**Table 1: Primer homology tail sequences for cloning homology arms into pEL226**

**Table:** 

**Primer homology tails** ​	**homology tail sequence** ​
​**forward primer 5’ homology (*SpeI*)**	​aaacgacggccagtgaattca
​**reverse primer 5’ homology ​(*SpeI*, introducing an alanine 5’ of *mCherry*)**	cttcttcaccctttgagaccatagc
​**forward primer 3’ homology ​(*NotI*, introducing an alanine 3’ of *fkbp12*, [optional stop codon])**	cgagctcctcaagctcgaggct[tag]​
**reverse primer 3’ homology (*NotI*)**	tgattacgccaagcttgcg

**Table** **2: Used *C. elegans* strain**

**Table:** 

Strain	Genotype	References
​**ZAN87​**	*estSi50[pEZ156;pmex-5::frb::gfp::ph::tbb2; Cbr-unc-119(+)]*I; *unc-119(ed3)* III	(Mangal *et al.* 2018)
**EJ1269**	*mex-6(dx203*[*mex-6::mcherry::fkbp12* + *loxP*]*)* II	This study
**EJ1270**	*estSi50[pEZ156;pmex-5::frb::gfp::ph::tbb2; Cbr-unc-119(+)]*I;*mex-6(dx203*[*mex-6::mcherry::fkbp12* + *loxP*]*)* II[tag]​	This study
