# Erratum: Establishment of a CRISPR/Cas9-based strategy for inducible protein dimerization

**DOI:** 10.17912/micropub.biology.000195

**Published:** 2019-12-02

## Description

Zeilich, J; Mangal, S; Zanin, E; Lambie, EJ (2018). Establishment of a CRISPR/Cas9-based strategy for inducible protein dimerization. microPublication Biology. 10.17912/W2208R published online April 17, 2018; corrected after publication Dec 2, 2019.

In the version of the microPublication originally published online, the last name of the first author was misspelled. The correct spelling is Zielich. This mistake has been corrected online. The spelling was correct in the original PDF version.

